# Association between cortisol and aging-related hippocampus volume changes in community-dwelling older adults: a 7-year follow-up study

**DOI:** 10.1186/s12877-022-03455-z

**Published:** 2022-09-21

**Authors:** Ryuzo Orihashi, Yoshiomi Imamura, Shigeto Yamada, Akira Monji, Yoshito Mizoguchi

**Affiliations:** 1 School of Nursing, Faculty of Medicine, Oita University, 1-1 Idaigaoka, Hasama-machi, Yufu, Oita 879-5593 Japan; 2 Department of Psychiatry, Faculty of Medicine, Saga University, 5-1-1 Nabeshima, Saga, 849-8501 Japan; 3 Institute of Comparative Studies of International Cultures and Societies, Kurume University, 1635 Mii-machi, Kurume, Fukuoka 839-8502 Japan; 4 St. Lucia’s Hospital, 1012 Tsubukumotomachi, Kurume, Fukuoka 830-0047 Japan

**Keywords:** Cognitive function, Cortisol, Hippocampus, MRI, Voxel-based morphometry

## Abstract

**Background:**

Identifying peripheral biomarkers related to modifiable risk factors to prevent dementia at an early stage will be extremely beneficial. We have been studying how older adults can maintain their mental health and continue to live in a familiar community. The aim of this study is to investigate the association between serum cortisol levels and brain volume among older adults in rural Japan.

**Methods:**

This was a longitudinal study conducted in Kurokawa-cho, Imari, Saga Prefecture, Japan, among people aged 65 years and above, as reported previously. We conducted a survey twice. The first survey was conducted from October 2009 to March 2011 (Timepoint 1) and the second was conducted from November 2016 to September 2017 (Timepoint 2). Blood samples for serum cortisol levels analysis were collected from participants at Timepoint 1. Serum cortisol levels were measured using the enzyme-linked immunosorbent assay. The participants underwent brain MRI examinations, and Mini-Mental State Examination (MMSE) and Clinical Dementia Rating (CDR) for cognitive function assessment at Timepoint 1 and Timepoint 2. We obtained 70 participants (16 men, mean age 72.69 ± 3.18 years; 54 women, mean age 72.69 ± 4.60 years, at Timepoint 1) for analysis. Correlation analysis was performed between serum cortisol levels at baseline (Timepoint 1) and brain volume (Timepoint 1, Timepoint 2, and Timepoint 1–Timepoint 2 difference) using voxel-based morphometry method.

**Results:**

There was no significant difference in serum cortisol levels between men (72.32 ± 17.30 ng/ml) and women (76.60 ± 21.12 ng/ml) at baseline. Additionally, no effect of blood collection time on cortisol levels was observed in these participants. Small volume correction analysis at the cluster level by applying multiple comparison corrections (family-wise error; P < 0.05) showed a negative correlation between serum cortisol levels (Timepoint 1) and brain volume (Timepoint 2) within the region containing the left hippocampus.

**Conclusions:**

Serum cortisol levels may serve as a peripheral biomarker of age-related volume changes involving the hippocampus in older adults aged 65 years and above.

**Supplementary Information:**

The online version contains supplementary material available at 10.1186/s12877-022-03455-z.

## Introduction

Dementia, including Alzheimer’s disease (AD), is a major global public health problem. According to Alzheimer’s Disease International, dementia affects more than 50 million people worldwide, with a new case of dementia occurring somewhere in the world every three seconds. A radical therapeutic medicine for dementia has not yet been put into practical use. The pathological changes in AD begin with deposited beta-amyloid peptide found within plaques about 20 years before the onset of symptoms, followed by hyperphosphorylated tau protein found in neurofibrillary tangles [1]. Modifiable risk factors for preventing dementia and AD have been identified [2, 3]. These factors include smoking cessation and proper treatment of lifestyle-related diseases, such as diabetes, hypertension, obesity, and depression. Identifying peripheral biomarkers related to modifiable risk factors to prevent dementia at an early stage will be extremely beneficial. Peripheral samples such as blood and saliva have the advantage of being less invasive when taken from participants. If some peripheral biomarkers in the healthy state of the older adults are associated with predicting future deterioration of mental health, such as cognitive function, it should contribute to maintaining the health of the older adults. We have been studying how older adults can maintain their mental health and continue to live in a familiar community. To date, we have conducted epidemiological studies in older adults living in rural communities [4–7]. Nabeta et al. reported that higher salivary cortisol levels were associated with a later depressive state in older healthy women. Additionally, we attempted to develop peripheral biomarkers related to modifiable risk factors to prevent dementia by analyzing the association between peripheral biomarkers and brain volumes [8, 9].

In this study, we focused on cortisol, a lipophilic steroid hormone produced within the adrenal cortex. Exposure to stress activates the hypothalamic–pituitary–adrenal axis, resulting in cortisol secretion from the adrenal cortex. For example, acute stress tasks, such as oral presentations, may increase cortisol levels [10]. Such acute stress responses may positively or negatively affect a person’s motivation and performance. However, chronic high cortisol levels associated with chronic stress may have adverse health effects. The hippocampus is vulnerable to the neurotoxic effects of excess glucocorticoid levels, and elevated cortisol levels may exert detrimental effects on cognition, contributing to AD pathology [11–14]. It has also been reported that higher cortisol levels in older adults reduce overall cognitive performance [15, 16]. Animal studies have indicated that chronic exposure to elevated glucocorticoid or chronic stress can lead to neuronal atrophy involving the hippocampus [17]. As these reports show, the association between cortisol and the hippocampus and cognitive function has been clarified. There are also reports on the association between cortisol levels and brain volume [18–22]. Additionally, high cortisol levels are associated with smaller hippocampal volumes [23]. Hippocampal volume has been robustly associated with memory performance and increased risk of dementia and is considered a reliable MRI biomarker for disease progression [24, 25]. Additionally, to the best of our knowledge, there have been no similar long-term follow-up longitudinal studies in older adults. Therefore, it is clinically valuable to address the association between serum cortisol levels and brain volume, especially in the hippocampus. If this 7-year longitudinal study revealed an association between serum cortisol levels and brain volume, especially involving the hippocampus, it might strengthen the evidence that serum cortisol levels are one of the peripheral biomarkers related to modifiable risk factors to prevent dementia in older adults. Currently, the stressors associated with the coronavirus disease 2019 (COVID-19) epidemic may not be measurable. Therefore, investigating cortisol, which is associated with stress, is also beneficial for maintaining the health of older adults.

This study aimed to evaluate serum cortisol levels in older adults living in a rural community and examine its relationship with brain volume using MRI. To address this, we designed a prospective cohort study in which healthy older adults without dementia were examined longitudinally for seven years.

## Materials and methods

### Participant characteristics

This was a longitudinal study conducted in Kurokawa-cho, Imari, Saga Prefecture, Japan, among people aged 65 years and above, as reported previously [4–9]. Kurokawa-cho is in the northwestern Saga Prefecture and is a rural town that is somewhat cut-off from urban areas. The area of the town is 26.48 km^2^. Its main industries are shipbuilding and primary industries.

In this study, we collected data from 596 community-dwelling older adults. These 596 participants comprised 71.8% of the population of Kurokawa-cho over 65 years of age. A survey was conducted twice. First, from October 2009 to March 2011, we conducted a baseline survey termed “Timepoint 1”; second, we re-conducted the study from November 2016 to September 2017 (Timepoint 2).

Most of the survey at Timepoint 1 was conducted as a part of the national survey to obtain data to calculate the prevalence of dementia in Japan [26]. Participants underwent screening assessments according to a predetermined systematic procedure (refer to Ikejima et al. for full details). We asked participants to undergo MRI examinations when further assessments were necessary for dementia. We used the Diagnostic and Statistical Manual of Mental Disorders, third edition revised, to diagnose dementia with reference to MRI findings. As an additional method in our study, participants who were determined as not likely to have dementia during the screening process also underwent MRI examinations on patient request. Thus, not all participants underwent MRI examinations during this period. As a result, 333 participants underwent MRI examinations at Timepoint 1. Seven years after conducting the first survey (Timepoint 1), we notified the investigation of Timepoint 2 to 277 participants who underwent MRI examinations and were not diagnosed with dementia during the Timepoint 1 survey. The Timepoint 2 survey was intended to follow up from the Timepoint 1 survey and not diagnose dementia. However, only 73 participants (of Timepoint 1) agreed to participate in the investigation at Timepoint 2. Of the 73 participants, none were diagnosed with dementia at Timepoint 1. Thus, 204 participants dropped out arbitrarily between Timepoint 1 and Timepoint 2. Therefore, we do not know the exact reason for dropping out. In addition, we did not visit them before they dropped out. To select participants for analysis, we excluded three participants, one with no MRI samples and two with a Mini-Mental State Examination (MMSE) score (Timepoint 1) of less than 24, from 73 participants. Consequently, we obtained 70 participants (16 men, mean age 72.69 ± 3.18 years; 54 women, mean age 72.69 ± 4.60 years, at Timepoint 1) for analysis.

### Cognitive function assessment

The MMSE is a simple screening index that estimates cognitive function [27]. The Clinical Dementia Rating (CDR) is used for dementia evaluation and severity staging [28, 29]. All participants underwent MMSE and CDR for cognitive function assessment at Timepoint 1 and Timepoint 2.

### Serum samples

Blood samples for serum cortisol levels analysis were collected from participants either between 9:00 and 12:00 (AM) or between 12:00 and 15:00 (PM) during Timepoint 1. On the same day, at Saga University, all samples were centrifuged. Next, the serum was extracted, transferred to a container, and immediately stored at -80 °C [8, 9].

### Evaluation of serum cortisol levels and other risk factors

Serum was thawed at room temperature. All samples were analyzed in duplicate. Serum cortisol levels were analyzed using a commercially available cortisol ELISA kit (R&D Systems, Minneapolis, MN, USA) according to the manufacturer’s instructions. The intra- and inter-assay coefficients of variation were 6.97% and 13.63%, respectively. The baseline survey also included hypertension and metabolic status assessments, such as body mass index (BMI) and recording the history of diabetes and dyslipidemia [8, 9].

### MRI acquisition

MRI examinations were performed at Timepoint 1 and Timepoint 2 using a 1.5 Tesla device (Excelart Vantage AGV; Canon Medical Systems, Otawara, Japan). Three-dimensional T1-weighted structural images were acquired for each participant using a field echo three-dimensional method (TR: 21 ms, TE: 5.5 ms, flip angle: 20°, field of view: 240 × 240 mm, matrix: 256 × 256, slice thickness: 1.5 mm, number of slices: 124). The examination conditions were kept the same for all participants and followed a standardized procedure [8, 9].

### Statistical analysis

Each participant’s data were analyzed and compared using a commercially available statistical package (JMP 15.2.0; SAS Institute, Cary, NC, USA). The mean values were compared using Welch’s t-test. Fisher’s exact test was used to compare the prevalence of hypertension, diabetes, dyslipidemia, and blood collection time. Multiple regression analysis was used to determine the effects of age, sex, hypertension, metabolic status, and blood collection time on serum cortisol levels. The Wilcoxon signed-rank test was used to compare the MMSE and CDR scores during Timepoint 1 and Timepoint 2. Regarding the relationship with serum cortisol levels, multiple regression analysis was performed for changes in MMSE, and logistic regression analysis was performed for changes in CDR. Statistical significance was set at *P* < 0.05.

### Preprocessing of the brain MRI and longitudinal voxel-based morphometry analysis

Brain MRI processing and analysis were conducted using voxel-based morphometry (VBM) [30] implemented with Statistical Parametric Mapping (SPM12; Wellcome Department of Cognitive Neurology, London, UK) in MATLAB R2016a (MathWorks, Natick, MA, USA). We used the same methodology described in a previous study [8, 9].

T1-weighted MR images were first segmented for gray and white matter using the segmentation procedures implemented in SPM12. The diffeomorphic anatomical registration through the exponentiated lie algebra (DARTEL) tool, described in SPM12, was used on the segmented gray matter and white matter images to construct a template for co-registration across participants [30, 31]. The segmented gray matter and white matter images were co-registered to the final DARTEL template, and local volumes were preserved by modulating the image intensity of each voxel by the Jacobian determinants of the deformation fields computed by DARTEL. The registered images were smoothed with a Gaussian kernel with a full width at half maximum of 8 mm and then transformed into the Montreal Neurological Institute (MNI) stereotactic space using affine and nonlinear spatial normalization implemented in SPM12. Preprocessing was performed using Araya Brain Imaging (Tokyo, Japan).

Gray matter images were used for the analysis. After preparing Timepoint 1 and Timepoint 2 images, Timepoint 1–Timepoint 2 difference images were created by subtracting the Timepoint 2 images from the Timepoint 1 images [32]. The correlation between serum cortisol levels at baseline (Timepoint 1) and brain volume (Timepoint 1, Timepoint 2, and Timepoint 1–Timepoint 2 difference) was evaluated using gray matter images and multiple regression design. Men and women were analyzed together, with age, sex, and handedness as covariates. Moreover, the total brain volume at Timepoint 1 and Timepoint 2 was used as a covariate during the respective time points. The masking toolbox was used to create mask images for analysis, and a multiple comparison correction (family-wise error) was performed. The initial voxel threshold was set to *p* = 0.001, uncorrected. Clusters were considered significant when they fell below the cluster-corrected P (family-wise error) value (= 0.05). Thus, analyses at the cluster level were performed to identify significant brain regions. After statistically significant brain regions were determined, the anatomical labels were identified using automated anatomical labeling corresponding to the space of the MNI standard coordinate system [33].

## Results

### Participant characteristics, serum cortisol levels, and MMSE and CDR scores

There was no significant difference in serum cortisol levels between men (72.32 ± 17.30 ng/ml) and women (76.60 ± 21.12 ng/ml) at baseline. Furthermore, the average interval between Timepoint 1 and Timepoint 2 brain MRI examinations was the same in men and women. Moreover, no sex differences were observed regarding the prevalence of other risk factors or blood collection time (Table 1). Although the variability of cortisol levels was observed during the day in serum [34], no effect of blood collection time on cortisol levels was observed in these participants (Table 2). Overall, participant MMSE scores declined, and CDR scores increased from Timepoint 1 to Timepoint 2 (Table 3). Moreover, we adjusted for age and sex and analyzed the association between serum cortisol levels (Timepoint 1) and changes in cognitive function assessment (Timepoint 1–Timepoint 2 difference). The results showed that MMSE was not associated (Table 4), but high serum cortisol levels were associated with changes in CDR (Table 5). Additionally, of the 596 participants at Timepoint 1, we show comparisons such as age and cognitive function for the 70 participants for the final analysis and the other 526 participants who were lost to follow up (Supplementary Table 1).

**Table 1:** Participant demographics

	Overall	Men	Women	Statistical significance
*N*	70	16	54	
Age (years, Timepoint 1), mean ± SD	72.69 ± 4.29	72.69 ± 3.18	72.69 ± 4.60	P = 0.998^a^
Cortisol (ng/ml, Timepoint 1), mean ± SD	75.62 ± 20.27	72.32 ± 17.30	76.60 ± 21.12	P = 0.416^a^
Education (years), mean ± SD	9.89 ± 1.70	10.81 ± 2.10	9.61 ± 1.47	P = 0.046^a^
MRI interval (years, Timepoint 1 to Timepoint 2), mean ± SD	6.88 ± 0.62	6.86 ± 0.64	6.88 ± 0.62	P = 0.928^a^
BMI (kg/m^2^), mean ± SD	23.90 ± 3.22	23.98 ± 2.73	23.87 ± 3.37	P = 0.902^a^
Hypertension, *n* (%)	29 (42.6)	6 (40.0)	23 (43.4)	P = 1.000^b^
Diabetes, *n* (%)	14 (20.3)	3 (18.8)	11 (20.8)	P = 1.000^b^
Dyslipidemia, *n* (%)	26 (37.7)	3 (18.8)	23 (43.4)	P = 0.087^b^
Blood collection time, *n* (%)				
AM	36 (51.4)	6 (37.5)	30 (55.6)	P = 0.260^b^
PM	34 (48.6)	10 (62.5)	24 (44.4)	

Missing data: BMI (*N* = 2), Hypertension (*N* = 2), Diabetes (*N* = 1), Dyslipidemia (*N* = 1)

The blood collection time was defined as “AM” for 9:00 to 12:00 collection and “PM” for 12:00 to 15:00 collection

^a^Welch’s t*-*test and

^b^Fisher’s exact test

*BMI* Body mass index

**Table 2:** Multiple regression analysis between serum cortisol levels (Timepoint 1) as the dependent variable with age, sex, BMI, hypertension, diabetes, dyslipidemia and blood collection time as the independent variables

	estimate	T	P	lower 95% CI	upper 95% CI	β
Age	0.15	0.25	0.80	-1.06	1.36	0.03
Sex (Women)	1.96	0.63	0.53	-4.24	8.17	0.08
BMI	-0.10	-0.13	0.90	-1.72	1.51	-0.02
Hypertension (Yes)	-2.87	-1.11	0.27	-8.07	2.33	-0.14
Diabetes (Yes)	6.66	2.08	0.04	0.24	13.07	0.27
Dyslipidemia (Yes)	-2.82	-1.00	0.32	-8.45	2.81	-0.14
Blood collection time (AM)	2.19	0.86	0.39	-2.89	7.27	0.11

R^2^ = 0.10

95% *CI* 95% Confidence interval, *β* standard partial regression coefficient

**Table 3:** MMSE and CDR scores at Timepoint 1 and Timepoint 2

	Timepoint 1	Timepoint 2	Statistical significance
MMSE, mean ± SD	28.44 ± 1.45	26.87 ± 3.16	*P* < 0.0001
CDR, *n* (%)			
0	67 (95.7)	63 (90.0)	*P* = 0.029
0.5	3 (4.3)	6 (8.6)	
1		1 (1.4)	

Wilcoxon signed-rank test

*MMSE* Mini-mental state examination, *CDR* Clinical dementia rating

**Table 4:** Multiple regression analysis with serum cortisol levels (Timepoint 1) as the independent variable and changes in MMSE (Timepoint 1–Timepoint 2 difference) as the dependent variable

serum cortisol levels (Timepoint 1)	estimate	P	lower 95% CI	upper 95% CI	R^2^
Model 1: Unadjusted	0.018	0.272	-0.014	0.050	0.02
Model 2: Adjusted for age	0.018	0.213	-0.011	0.047	0.21
Model 3: Adjusted for age and sex	0.019	0.203	-0.010	0.048	0.21

95% *CI* 95% Confidence interval

**Table 5:** Logistic regression analysis with serum cortisol levels (Timepoint 1) as the independent variable and changes (yes/no) in CDR (Timepoint 1–Timepoint 2 difference) as the dependent variable

serum cortisol levels (Timepoint 1)	OR	P	lower 95% CI	upper 95% CI	R^2^
Model 1: Unadjusted	1.098	0.010	1.023	1.179	0.36
Model 2: Adjusted for age	1.103	0.016	1.019	1.195	0.47
Model 3: Adjusted for age and sex	1.099	0.020	1.015	1.191	0.50

95% *CI* 95% Confidence interval, *OR* Odds ratio

### Voxel-based morphometry findings

**Fig. 1:**
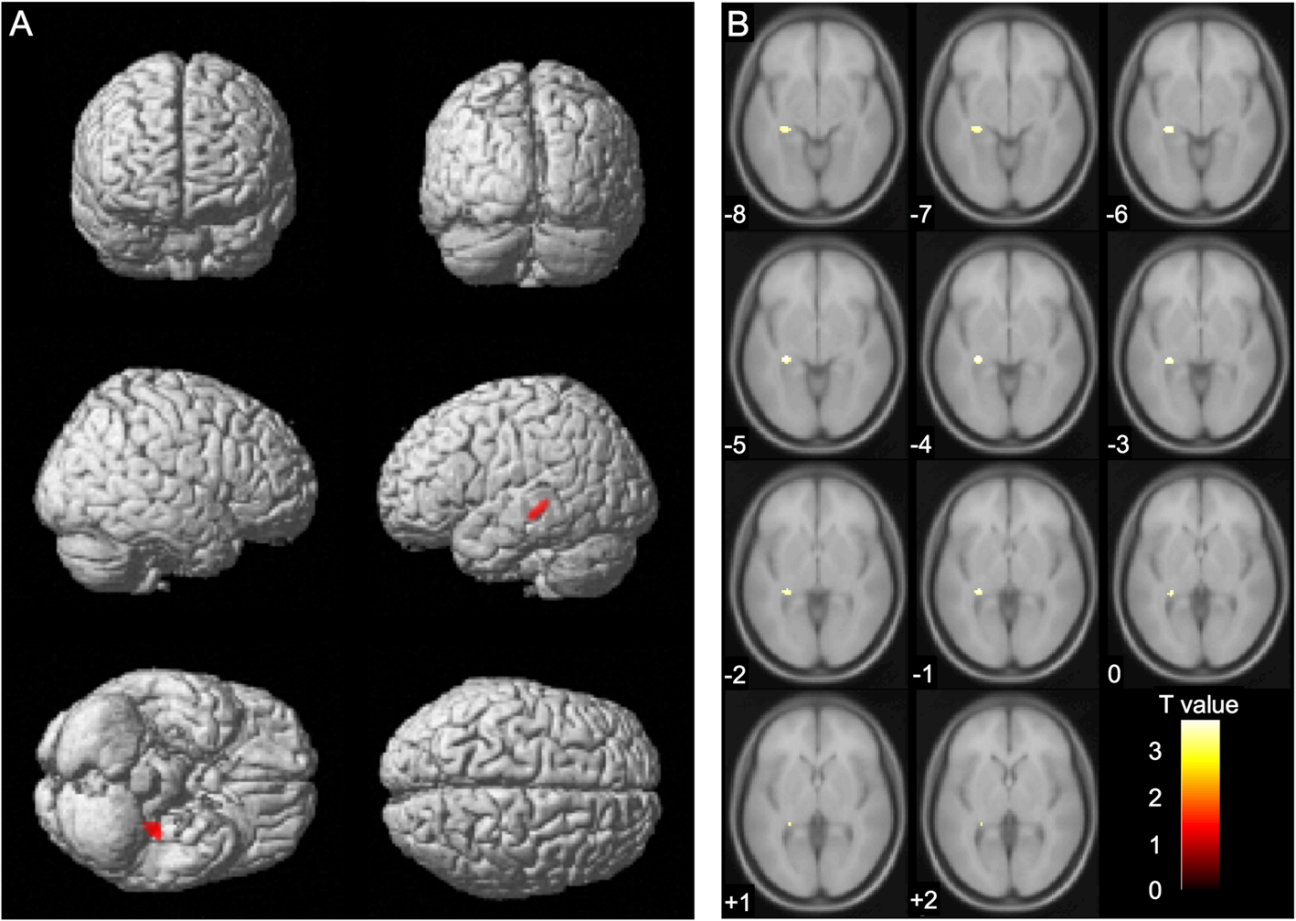
Voxel-based morphometry findings: Association between serum cortisol levels (Timepoint 1) and brain volume (Timepoint 2). Multiple regression analysis showed a negative correlation between serum cortisol levels (Timepoint 1) and brain volume (Timepoint 2). The threshold for statistics was set to T = 3.22 for the height threshold and k = 122 voxels for the extent threshold. The significant cluster containing the left hippocampus (coordinates -30, -30, -5) is shown in (**A**) whole-brain images and (**B**) axial images. The T value is applied to the axial images

**Fig. 2:**
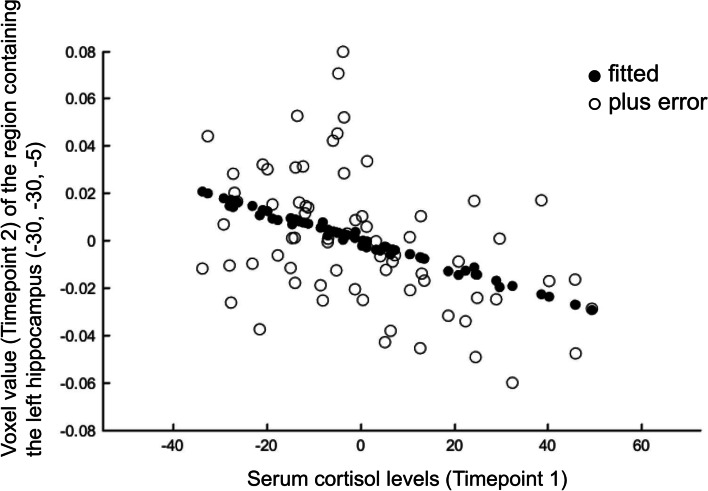
Correlation between serum cortisol levels and voxel values. Scatterplot of serum cortisol levels (Timepoint 1) and voxel values (Timepoint 2) of the region containing the left hippocampus (coordinates -30, -30, -5). The horizontal axis plots serum cortisol levels – averaged value. Zero on the horizontal and vertical axis represent the average value, respectively. The plots on the vertical axis are as follows. Fitted value = voxel value – (average voxel value + error). Plus error = voxel value – averaged voxel value

Correlation analysis was performed between serum cortisol levels at baseline (Timepoint 1) and brain volume (Timepoint 1, Timepoint 2, and Timepoint 1–Timepoint 2 difference) using voxel-based morphometry method [8, 9]. However, in any cases, there was no correlation between serum cortisol levels and brain volume at the cluster level analysis (family-wise error; *P* < 0.05). Therefore, we focused on the results of the uncorrected analysis at the peak level (*P* < 0.001). The results showed that there was a negative correlation between serum cortisol levels (Timepoint 1) and brain volume (Timepoint 2) of the region containing the left hippocampus (coordinates -30, -30, -5). The expected voxels per cluster was 121.736, and the threshold for statistics was set to T = 3.22 for the height threshold and k = 122 voxels for the extent threshold. Based on the results, the cluster containing the left hippocampus was set as the region of interest. Subsequently, a small volume correction analysis at the cluster level was performed by applying multiple comparison corrections (family-wise error; *P* < 0.05) [35]. When performing small volume correction analysis, a sphere with a radius of 10.0 mm was set as the region of interest centered on the peak coordinates (-30, -30, -5) of this cluster. The small volume correction analysis results are shown in Table 6 with details regarding P, T, and cluster size (number of voxels) values. The VBM findings on the significant cluster containing the left hippocampus are shown in Fig. 1, using standard brain MR images. Furthermore, a scatterplot of serum cortisol levels (Timepoint 1) and voxel values (Timepoint 2) of the region containing the left hippocampus (coordinates -30, -30, -5) are shown in Fig. 2.

**Table 6:** Voxel-based morphometry finding. Negative correlation between serum cortisol levels (Timepoint 1) and brain volume (Timepoint 2) by multiple regression analysis

cluster-level		peak-level		MNI coordinates				
P FWE-corr	k, cluster size (voxels)	T	P uncorr	X(mm)	Y(mm)	Z(mm)		anatomical region
0.008	131	3.68	< 0.001	-30	-30	-5		Left hippocampus

Height threshold, T = 3.22. Extent threshold, k = 122 voxels. Expected voxels per cluster, k = 121.736

Degrees of freedom = [1.0, 64.0]. FWHM = 16.1, 15.9, and 15.2 mm; 10.7, 10.6, and 10.1 voxels

Volume, 2039 = 604 voxels = 1.1 resels. Voxel size, 1.5 mm × 1.5 mm × 1.5 mm (resel = 1151.25 voxels)

*FWE* Family-wise error, *corr* corrected, *uncorr* uncorrected, *MNI* Montreal neurological institute, *FWHM* Full width at half maximum

Labels are marked using automated anatomical labeling

## Discussion

We evaluated serum cortisol levels in older adults and examined its relationship with brain volume using MRI [8, 9]. There was no correlation between serum cortisol levels and brain volume at the cluster level analysis (family-wise error; *P* < 0.05), either cross-sectionally or longitudinally. However, when the small volume correction was analyzed, we observed that baseline serum cortisol levels correlated negatively with left hippocampal volume seven years later. Additionally, high serum cortisol levels were associated with changes in CDR.

In analysis targeting the whole brain, the number of voxels to be tested increases [8, 9]. Therefore, when applying the multiple comparison correction, it may be a conservative method that makes it difficult to obtain significant conclusions. In small volume correction analysis, the number of voxels to be tested is smaller than in whole brain analysis, and significant conclusions may be obtained in some cases [35]. Based on previous reports [23], we hypothesized that there is an association between serum cortisol levels and hippocampal volume. Similarly, if any hypothesis about the association between other biomarkers and brain volume can be considered, it may be worth considering a small volume correction analysis even if the whole-brain analysis does not yield significant conclusions. Moreover, it may be important to ensure a sufficient sample size and adjust for possible confounding factors in order to obtain significant conclusions. Cortisol seems to be among the hormones with the most important effects on brain function. Additionally, as is well known, the hippocampus is closely associated with memory and cognitive function [36, 37]. Hippocampal volume has been robustly associated with memory performance and the risk of dementia and is considered a reliable MRI biomarker for disease progression [24, 25]. As far as we know, there were no longitudinal studies in older adults with a follow-up period of about seven years or longer. In the people aged over 65 years in our study, serum cortisol levels in cognitively healthy conditions were negatively correlated with hippocampal volume after seven years. Based on many studies to date, our results may strengthen the evidence that serum cortisol levels are one of the biomarkers related to modifiable risk factors to prevent dementia in older adults.

In our previous studies involving whole-brain analysis, we observed that serum oxytocin levels in older adults were positively correlated with future hippocampal and amygdala volumes [8]. However, we did not observe a correlation with serum soluble triggering receptor expressed on myeloid cells 2 levels [9]. Therefore, we consider serum cortisol levels to be as important a peripheral biomarker as oxytocin in relation to hippocampus volume and cognitive function. Certainly, atrophy or deterioration of the brain, including the hippocampus, may occur due to aging [38]. Indeed, reports on hippocampal volume in older adults have shown that patients with AD have significantly smaller hippocampal volumes than healthy controls [39, 40]. From these findings, for older adults aged 65 years and above, avoiding chronically high cortisol levels seem to be one of the factors in maintaining a cognitively healthy life. Chronic stress, sleep, BMI, metabolic syndrome, and depression have been shown to be associated with cortisol levels [41–46]. Many of these are also correctable risk factors for preventing the development of dementia and AD [2, 3]. Based on these facts, we believe that health education and health counseling for the prevention of dementia by medical professionals with specialized knowledge, such as doctors and nurses, may be effective in the community. This study showed an association between serum cortisol levels, hippocampal volume, and cognitive function. It can be inferred that reducing stress in daily life and improving the lifestyle as needed may lead to cognitive health in older adults. We hope that the results of our research will lead to self-care behavior for preventing cognitive decline and aging in older adults in the future.

There are limitations that need to be considered with regard to this current study. As mentioned in the Materials and Methods section, not all participants underwent MRI examinations at Timepoint 1, and the cohort may be biased. There were significant differences in those included in the manuscript and those loss to follow up in age, education, MMSE and CDR. The p-values were very strongly significant (Supplementary Table 1). Some of this attrition may likely be due to healthy cohort bias. Therefore, the cohort may not reflect the characteristics of a rural older adult population in Japan. Only MMSE and CDR were used to assess cognitive function. We did not obtain any information related to dementia, such as smoking history, sleep disorders, and lifestyle. Additionally, our study was limited by the high number of individuals who dropped out during Timepoint 1 and Timepoint 2. Furthermore, due to small sample size, possible confounding factors could not be sufficiently adjusted when analyzing the correlation between serum cortisol levels and brain volume.

In conclusion, we focused on the correlation between serum cortisol levels and brain volume in older adults living in a rural community. We found that, for people aged over 65 years, serum cortisol levels correlated negatively with left hippocampal volume after seven years. This result suggests that serum cortisol levels may serve as a peripheral biomarker of age-related volume changes involving the hippocampus in older adults aged 65 years and above.

## Supplementary Information

**Additional file 1:** **Supplementary Table 1.** The 596 participants demographics at Timepoint 1. The 70 participants for the final analysis and the other 526 participants.

## Data Availability

The data that support the findings of this study are available from the corresponding author (Yoshito Mizoguchi) but restrictions apply to the availability of these data, due to the restriction under the institutional ethical committee’s policy and so are not publicly available. Data are however available from the authors upon reasonable request and with permission of the corresponding author (Yoshito Mizoguchi).

## Abbreviations

AD: Alzheimer’s disease
BMI: Body mass index
CDR: Clinical dementia rating
DARTEL: Diffeomorphic anatomical registration through exponentiated lie algebra
MMSE: Mini-mental state examination
MNI: Montreal neurological institute
SPM: Statistical parametric mapping
VBM: Voxel-based morphometry
