# Supplementary material for: Association between cortisol and aging-related hippocampus volume changes in community-dwelling older adults: a 7-year follow-up study
Source: BMC Geriatr. 2022 Sep 21;22:765. doi: 10.1186/s12877-022-03455-z (PMC9491648; doi:10.1186/s12877-022-03455-z)
Supplement: Supplementary file 1 — Additional file 1: Supplementary Table 1. The 596 participants demographics at Timepoint 1. The 70 participants for the final analysis and the other 526 participants. [file 12877_2022_3455_MOESM1_ESM.pdf]

Supplementary Table 1

The 596 participants demographics at Timepoint 1. The 70 participants for the final analysis and the other 526 participants.

N	70 (16 men, 54 women)	526 (200 men, 326 women)	Statistical significance
Age (years, Timepoint 1), mean ± SD	72.69 ± 4.29	77.29 ± 7.36	P < 0.0001[a]
Education (years), mean ± SD	9.89 ± 1.70	9.05 ± 1.92	P = 0.0003[a]
BMI (kg/m^2), mean ± SD	23.90 ± 3.22	23.26 ± 3.40	P = 0.133[a]
MMSE (Timepoint 1), mean ± SD	28.44 ± 1.45	25.23 ± 6.68	P < 0.0001[a]
CDR (Timepoint 1), *n* (%)			
0	67 (95.7)	374 (71.1)	
0.5	3 (4.3)	86 (16.3)	
1		20 (3.8)	
2		21 (4.0)	
3		25 (4.8)	
0.5 or more	3 (4.3)	152 (28.9)	P < 0.0001[b]

Missing data: Education (N = 27), BMI (N = 62), MMSE (N = 5).

[a]Welch’s t-test and [b]Fisher’s exact test.

BMI = body mass index; MMSE = Mini-Mental State Examination; CDR = Clinical Dementia Rating.
